# Positive Epstein–Barr virus detection in coronavirus disease 2019 (COVID-19) patients

**DOI:** 10.1038/s41598-021-90351-y

**Published:** 2021-05-25

**Authors:** Ting Chen, Jiayi Song, Hongli Liu, Hongmei Zheng, Changzheng Chen

**Affiliations:** 1 Renmin Hospital of Wuhan University, Wuhan, 430060 Hubei China; 2 Department of Ophthalmology, Renmin Hospital of Wuhan University, No. 238 JieFang Road, Wuchang District, Wuhan, 430060 Hubei China

**Keywords:** Infectious-disease diagnostics, SARS-CoV-2, Viral infection

## Abstract

The objective of this study was to detect the Epstein–Barr virus (EBV) coinfection in coronavirus disease 2019 (COVID-19). In this retrospective single-center study, we included 67 COVID-19 patients with onset time within 2 weeks in Renmin Hospital of Wuhan University from January 9 to February 29, 2020. Patients were divided into EBV/SARS-CoV-2 coinfection group and SARS-CoV-2 infection alone group according to the serological results of EBV, and the characteristics differences between the two groups were compared. The median age was 37 years, with 35 (52.2%) females. Among these COVID-19 patients, thirty-seven (55.2%) patients were seropositive for EBV viral capsid antigen (VCA) IgM antibody. EBV/SARS-CoV-2 coinfection patients had a 3.09-fold risk of having a fever symptom than SARS-CoV-2 infection alone patients (95% CI 1.11–8.56; P = 0.03). C-reactive protein (CRP) (P = 0.02) and the aspartate aminotransferase (AST) (P = 0.04) in EBV/SARS-CoV-2 coinfection patients were higher than that in SARS-CoV-2 infection alone patients. EBV/SARS-CoV-2 coinfection patients had a higher portion of corticosteroid use than the SARS-CoV-2 infection alone patients (P = 0.03). We find a high incidence of EBV coinfection in COVID-19 patients. EBV/SARS-CoV-2 coinfection was associated with fever and increased inflammation. EBV reactivation may associated with the severity of COVID-19.

## Introduction

Since December 2019, a novel coronavirus named severe acute respiratory syndrome coronavirus 2 (SARS-CoV-2) caused an outbreak of coronavirus disease 2019 (COVID-19) in Wuhan, China^1,2^. SARS-CoV-2 was highly contagious and has rapidly spread. Therefore, World Health Organization (WHO) made the assessment that "COVID-19 could be characterized as a pandemic and called every day for countries to take urgent and aggressive action"^3^. COVID-19 developed rapidly, according to the report, the median time from symptom onset to ICU admission was 9.5 days, and the median time from ICU admission to death was 7 days^4^. Unfortunately, there was no proven effective treatment for coronavirus except for supportive care^5^.

In order to determine the source of infection and find more truth about the unexplained pneumonia, multiple virus tests were performed on our COVID-19 patients. According to the laboratory results of COVID-19 patients, it was noted that some patients were positive for Epstein–Barr virus (EBV) viral capsid antigen (VCA) IgM antibody. Although the infection rate of EBV is up to 90% in the adult population, most immunocompetent people have no clinical manifestations after infection^6^. However, it can be reactivated and proliferated in immunocompromised individuals, with fatal outcome^7^. EBV infection have been reported in some carcinomas such as Burkitt lymphoma, nasopharyngeal carcinomas and T-cell/NK lymphoma, as well as autoimmune diseases including systemic lupus erythematosus (SLE) and multiple sclerosis (MS)^8–10^. Recently, the pathological report of COVID-19 dead patient suggested the overactivation of T cells, suggesting a severe immune injury in COVID-19 patients^11^. Moreover, the similar symptoms such as fever, fatigue, myalgia, anorexia and sore throat between COVID-19^12,13^ and EBV-induced infectious mononucleosis (IM) indicated a potential association. So we hypothesized that there may be EBV coinfection in COVID-19 patients. In this study, we described the clinical characteristics of patients with confirmed SARS-CoV-2 infection, and compared the differences between EBV/SARS-CoV-2 coinfection patients and SARS-CoV-2 infection alone patients, so as to find out whether EBV/SARS-CoV-2 coinfection affects the disease progression and give a clue to clinical judgment.

## Results

### Patients characteristics

**Figure 1:**
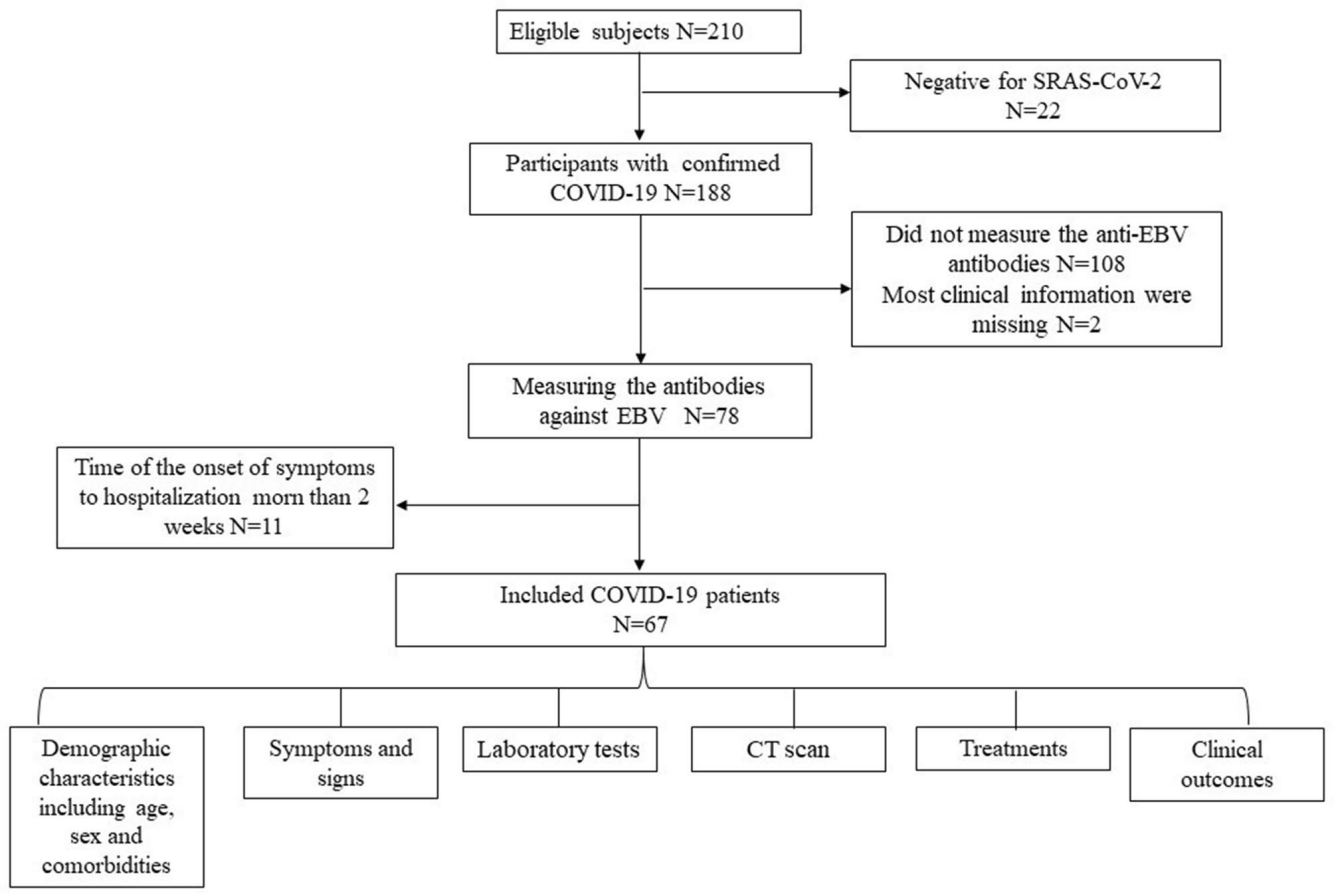
The workflow of corona virus disease 2019 (COVID-19) patients’ inclusion and exclusion criteria.

Figure 1 showed the workflow of COVID-19 patients’ inclusion and exclusion criteria. Among 210 hospitalized patients from January 9 to February 29, 2020, 188 patients had positive SARS-CoV-2 RT-PCR results. According to the inclusion and exclusion criterion, Sixty-seven COVID-19 patients having the results of anti-EBV antibodies were included to the final analysis. The median age of these COVID-19 patients was 37 years (IQR 30–52; range 23–81 years), with 35 (52.2%) were females. The median durations from onset of the first symptom to hospitalization was 4 days (IQR 3–7 days) (Table 1). Among the 67 COVID-19 patients, 11 (16.4%) had 1 or more combined diseases, as follows, cardiovascular disease (4 [6.0%]), hypertension (4 [6.0%]), diabetes (3 [4.5%]) and chronic liver disease (2 [3.0%]) and digestive system disease (1 [1.5%]).

**Table 1:** Characteristics of Epstein–Barr virus (EBV)/severe acute respiratory syndrome coronavirus 2(SARS-CoV-2) coinfection and SARS-CoV-2 infection alone patients.

	Total (n = 67)	VCA IgM antibody		
		Positive (n = 37)	Negative (n = 30)	P value
Age, years	37 (30–52)	36 (28–52)	37 (31–52)	0.64
Female	35 (52.2)	17 (46.0)	18 (60.0)	0.25
Onset of symptom to hospital admission	4 (3–7)	4 (3–7)	4 (2–7)	0.94
Combined diseases	11 (16.4)	8 (21.6)	3 (10.0)	0.34
**Symptoms**				
Fever	41 (61.2)	27 (73.0)	14 (46.7)	0.03
Dry cough	35 (52.2)	23 (62.2)	12 (40.0)	0.07
Fatigue	31 (46.3)	14 (37.8)	17 (56.7)	0.12
Anorexia	16 (23.9)	11 (29.7)	5 (16.7)	0.21
Myalgia	18 (26.9)	11 (29.7)	7 (23.3)	0.56
Sore throat	11 (16.4)	5 (13.5)	6 (20.0)	0.70
Expectoration	11 (16.4)	7 (18.9)	4 (13.3)	0.78
Chest congestion	10 (14.9)	7 (18.9)	3 (10.0)	0.50
**Vital signs**				
Heart rate, bpm	78 (71–82)	78 (68–80)	78 (74–86)	0.51
Respiratory, bpm	19 (18–20)	18 (18–20)	19 (18–20)	0.42
Mean arterial pressure, mmHg	88 (86–92)	88 (86–92)	89 (86–94)	0.46
Temperature, °C	36.6 (36.5–37.0)	36.6 (36.5–37.0)	36.6 (36.5–37.0)	0.54

The most common initial symptoms were fever (41 [61.2%]), dry cough (35 [52.2%]), fatigue (31 [46.3%]), myalgia (18 [26.9%]) and anorexia (16 [23.9%]). Other symptoms such as sore throat, expectoration and chest congestion were less common (Table 1). CT abnormality was found in 63 (94.0%) COVID-19 patients, and ground-glass opacity (45 [72.6%]) was the commonest manifestation.

Among these COVID-19 patients, 37 (55.2%) patients were seropositive for anti-VCA IgM, 63 (94.0%) were seropositive for anti-VCA IgG and 64 (95.5%) were seropositive for anti-EBNA IgG. There were 36 (53.7%) patients were seropositive for anti-VCA IgM + anti-VCA IgG + anti-EBNA IgG + anti-EA IgM- or anti-VCA IgM + anti-VCA IgG + anti-EBNA IgG + anti-EA IgM or anti-VCA IgM + anti-EBNA IgG − anti-EBNA IgG + anti-EA IgM-, which indicated the recovery/reactivation of the EBV infection^14^.

About other co-infection pathogens investigated in our study, only 8.1% (5/62) COVID-19 patients had positive anti-MP IgM and 1.6% (1/62) were positive for anti-RSV IgM. Among the patients with positive anti-MP IgM, 2 did the PCR Capillary Electrophoresis Fragment Analysis for MP, with negative result. 13 respiratory viruses were all negative in the tested 54 COVID-19 patients. The median CMV IgM antibody was 0.07 (IQR 0.04–0.18; normal range 0–12) AU/mL and CMV IgG antibody was 874.11 (IQR 341.11–1518.74; normal range 0–14) AU/mL in 59 COVID-19 patients.

### EBV/SARS-CoV-2 coinfection vs SARS-CoV-2 infection alone

When clinical symptoms in COVID-19 patients were compared with EBV VCA IgM antibody, EBV/SARS-CoV-2 coinfection patients had a higher risk to report fever symptom than SARS-CoV-2 infection alone patients (OR, 3.09; 95% CI 1.11–8.56; P = 0.03). There were no significant association between any other clinical symptoms, such as dry cough, fatigue, anorexia, myalgia and sore throat and EBV VCA IgM antibody in COVID-19 patients (Table 1). The key laboratory parameters in EBV/SARS-CoV-2 coinfection patients and SARS-CoV-2 infection alone patients were shown in Table 2. The aspartate aminotransferase (AST) in EBV/SARS-CoV-2 coinfection patients were significantly higher than that in SARS-CoV-2 infection alone patients (P = 0.04). No other significant differences were detected between EBV/SARS-CoV-2 coinfection and SARS-CoV-2 infection alone patients in blood routine examination and blood biochemistry results. C-reactive protein (CRP) in EBV/SARS-CoV-2 coinfection patients were higher than that in SARS-CoV-2 infection alone patients (P = 0.02). There were no statistically significant differences in humoral immunity parameters between EBV/SARS-CoV-2 coinfection and SARS-CoV-2 infection alone patients. The values of humoral immunity parameters in all COVID-19 patients were all in the normal range. Although the CD8 count was lower in EBV/SARS-CoV-2 coinfection patients than that in SARS-CoV-2 infection alone patients, the difference was not significant (P = 0.07). No statistically significant differences existed between cellular immunity parameters and the EBV VCA IgM antibody. The median counts of CD3, CD4, CD8, CD19 and CD16 + 56 were all in the normal range.

**Table 2:** Laboratory findings of Epstein–Barr virus (EBV)/severe acute respiratory syndrome coronavirus 2(SARS-CoV-2) coinfection and SARS-CoV-2 infection alone patients.

	Normal range	Total (n = 62)	VCA IgM antibody		
			Positive (n = 35)	Negative (n = 27)	P value
**Blood routine**					
White blood cell count, × 10^9^/L	3.5–9.5	4.27 (3.50–5.44)	4.10 (3.33–4.99)	4.53 (3.70–5.61)	0.11
Neutrophil count, × 10^9^/L	1.8–6.3	2.38 (1.85–3.23)	2.21 (1.65–2.89)	2.59 (2.16–3.42)	0.11
Lymphocyte count, × 10^9^/L	1.1–3.2	1.21 (0.98–1.64)	1.19 (0.98–1.73)	1.40 (0.99–1.52)	0.78
Monocyte count, × 10^9^/L	0.1–0.6	0.43 (0.31–0.52)	0.41 (0.35–0.47)	0.45 (0.30–0.57)	0.42
Red blood cell, × 10^12^/L	3.8–5.8	4.46 (4.16–4.85)	4.44 (4.13–4.82)	4.47 (4.18–4.85)	0.69
Platelet count, × 10^9^/L	125–350	177 (147–227)	161 (145–203)	195 (165–237)	0.06
**Blood biochemistry**					
Alanine aminotransferase, U/L	7–50	18 (12–31)	24 (14–39)	17.5 (11–25)	0.12
Aspartate aminotransferase, U/L	13–40	22 (18 -28)	24 (19–30)	20.5 (17–24)	0.04
Total bilirubin, umol/L	0–23	8.3 (6.1–12.0)	8.6 (6.6–10.7)	8.3 (6.1–12.8)	0.73
Creatinine, μmol/L	41–97	59 (49–72)	63 (51–72)	53.5 (48–69)	0.42
Blood urea nitrogen, mmol/L	2.6–8.0	4.15 (3.52–4.71)	4.19 (3.73–4.72)	3.95 (3.37–4.63)	0.52
Potassium, mmol/L	3.5–5.3	4.08 (3.88–4.37)	4.08 (3.84–4.30)	4.10 (3.91–4.39)	0.61
Creatine kinase, U/L	40–310	73.5 (49.0–99.5)	75 (47–101)	67 (51–90)	0.33
Lactate dehydrogenase, U/L	120–250	193 (171–234)	204 (175–265)	185.5 (169.5–217.5)	0.33
Glucose, mmol/L	3.9–6.1	4.86 (4.52–5.54)	4.86 (4.61–5.54)	4.83 (4.49–5.46)	0.50
**Infection-related biomarkers**					
C-reactive protein, mg/L	0–10	4.85 (0.5–17.4)	8.2 (0.5–24.7)	2.0 (0.5–5.7)	0.02
**Humoral immunity**					
Serum IgG, g/L	8–16	11.10 (9.89–13.10)	10.90 (9.89–13.1)	11.20 (10.20–12.60)	0.96
Serum IgM, g/L	0.4–3.45	0.959 (0.688–1.290)	1.030 (0.688–1.440)	0.924 (0.722–1.150)	0.46
Serum IgA, g/L	0.76–3.9	1.93 (1.54–2.72)	1.94 (1.54–2.72)	1.92 (1.60–2.64)	0.86
C3, g/L	0.81–1.6	0.879 (0.740–0.983)	0.878 (0.738–0.988)	0.890 (0.774–0.963)	0.69
C4, g/L	0.1–0.4	0.260 (0.213–0.320)	0.266 (0.213–0.321)	0.243 (0.216–0.319)	0.52
**Cellular immunity**					
CD3 count, /uL	723–2737	752 (589–1047)	746 (569–1006)	871 (669–1047)	0.34
CD4 count, /uL	404–1612	429.5 (308–565)	406 (308–628)	469 (366–545)	0.57
CD8 count, /uL	220–1129	276.5 (194–424)	254 (188–350)	310 (235–480)	0.07
CD19 count, /uL	80–616	137.5 (103–179)	139 (98–186)	136 (114–168)	0.97
CD16 + 56 count, /uL	84–724	155 (97–274)	154 (86–275)	156 (115–248)	0.99
CD4/CD8, ratio	0.9–2.0	1.56 (1.14–2.12)	1.64 (1.24–2.18)	1.51 (1.07–1.66)	0.10

Sixty (89.6%) patients received interferon alpha inhalation, fifty-nine (88.1%) patients were given empirical antibiotic treatment, thirty-seven (55.2%) were given antiviral treatment, thirty-two (47.8%) patients received systematic corticosteroid treatment and 28 (41.8%) patients were given gamma globulin therapy (Table 3). Ten (14.9%) patients went to the ICU, of which seven (18.9%) patients were in the EBV/SARS-CoV-2 coinfection group. There was no statistical significance in the prevalence of patients going to the ICU (P = 0.30). No patients died in our study. EBV/SARS-CoV-2 coinfection patients had a higher portion of corticosteroid use than the SARS-CoV-2 infection alone patients (P = 0.03). Eight (11.9%) patients received oxygen inhalation, EBV/SARS-CoV-2 coinfection patients had a higher portion of oxygen inhalation than the SARS-CoV-2 infection alone patients while the difference was not significant (P = 0.11) (Table 3). The median recovery time for COVID-19 patients was 34 days (IQR 23–42 days), with 36 days for EBV/SARS-CoV-2 coinfection patients (IQR 25–42 days) and 34 days for SARS-CoV-2 infection alone patients (IQR 21–42 days). About the recovery time, the difference was not significant (P = 0.35).

**Table 3:** Treatments of Epstein–Barr virus (EBV)/severe acute respiratory syndrome coronavirus 2(SARS-CoV-2) coinfection and SARS-CoV-2 infection alone patients.

	Total (n = 67), n (%)	VCA IgM antibody		
		Positive (n = 37), n (%)	Negative (n = 30), n (%)	P value
Antiviral	37 (55.2)	22 (59.5)	15 (50.0)	0.44
Antibiotics	59 (88.1)	32 (86.5)	27 (90.0)	0.95
Corticosteroid	32 (47.8)	22 (59.5)	10 (33.3)	0.03
Gamma globulin	28 (41.8)	19 (51.4)	9 (30.0)	0.08
Interferon alpha inhalation	60 (89.6)	34 (91.9)	26 (86.7)	0.77
Oxygen inhalation	8 (11.9)	7 (18.9)	1 (3.3)	0.11

## Discussion

In this study, we described the clinical characteristics of COVID-19 patients, reported the EBV/SARS-CoV-2 coinfection and evaluated the clinical immune function to detect the possible mechanism for different clinical characteristics in COVID-19 patients. The main findings in our study were as follows: (1) more than half of COVID-19 patients were positive for EBV VCA IgM antibody; (2) EBV VCA IgM antibody was associated with fever, higher CRP and higher AST; (3) the EBV/SARS-CoV-2 coinfection patients were more likely to be given corticosteroid therapy by doctors; (4) The CD8 count in EBV/SARS-CoV-2 coinfection patients was a litter less than that in SARS-CoV-2 infection alone patients.

EBV is a ubiquitous human virus with a productive lytic cycle and a latent phase. The acute infection phase is mainly asymptomatic in children and the latent infection phase can be last for the whole life^15^. After EBV infection, specific antibodies are induced, including VCA IgM, IgG, EBNA IgG and EA IgM, IgG. The products in lytic infection phase include the EA complex and VCA. Serum positive for anti-VCA IgM indicates an acute infection, VCA IgG antibody appears at the acute infection stage, remaining positive for life, and EBNA IgG antibody is an indication of past infection^15^. Latent EBV can be reactivated and become a lytic infection, expressing anti-VCA IgM^16^. In our study, 55.2% COVID-19 patients had positive VCA IgM antibody, indicating a high incidence of EBV coinfection in COVID-19 patients. The VCA IgM antibody generally disappeared 1–2 weeks after onset^15^, and as a retrospective study, we could not confirm the times of EBV infection and SRAS-CoV-2 infection. To reduce the possibility of false negative VCA IgM antibody, we only included COVID-19 patients with onset time within 2 weeks. Meanwhile, the specificity of positive VCA IgM antibody need to be verified, as it may have cross-reactivities with CMV and other respiratory pathogens. Negative CMV IgM antibody was found in COVID-19 patients in our study. Other respiratory pathogens were also tested, only 8.1% COVID-19 patients had positive anti-MP IgM and 1.6% were positive for anti-RSV IgM. In the meantime, 2 COVID-19 patients had PCR Capillary Electrophoresis Fragment Analysis for the MP with negative result. Lehner et al.^17^ also observed that 78% of COVID-19 patients in the intensive care unit (ICU) had EBV viremia, and 39% even above 1000 IU/ml. Moreover, the prevalence and levels of EBV viremia in COVID-19 patients were significantly higher than those in non-COVID-19 patients. Thus, the possibility of false positive about the EBV coinfection in our study is small.

EBV reactivation has been reported in psychological stress of various type because of the impaired cellular immune function, including student examination stress^18^, attachment anxiety^19^ and loneliness^20^. During lytic stage of EBV infection, CD8 + T cells dominant the response for EBV infection^21^. Liu et al. found a decrease in CD8 count in the laboratory examination of 12 COVID-19 patients^22^. Paolucci et al.^23^ found a correlation between reduced CD8 + T cells and EBV DNA levels and COVID-19 severity. In our study, we also noted that CD8 count was lower in our EBV/SARS-CoV-2 coinfection patients, which neared statistical significance (P = 0.07). As our patients were adult population, therefore, we speculate that during acute viral infection (i.e., with SARS-CoV-2 or other viruses), a declining CD8 count may result in reactivation of EBV/EBV viraemia.

CRP, as an acute reactant, is produced in bacterial infection or inflammation^24^. Some studies reported that CRP was higher in the severe group than in the non-severe group^25,26^, and may also be a potential predictor of disease severity^27^. Other studies reported that cytokine storms might occur in COVID-19 patients, and the pro-inflammatory cytokine Th1, Th2 and Th17 were elevated^28^. In our study, the CRP in the EBV/SARS-CoV-2 coinfection patients were higher than that in the SARS-CoV-2 infection alone patients, indicating a powerful inflammatory response in EBV/SARS-CoV-2 coinfection patients. Meanwhile, EBV/SARS-CoV-2 coinfection patients had higher AST levels than SARS-CoV-2 infection alone patients in our study. Zhao et al.^29^ reported a higher levels of AST was found in COVID-19 patients when compared to non-COVID-19 pneumonia patients. Higher levels of AST and CRP were also found in refractory patients compared with general COVID-19 patients^30^. EBV DNA detection is frequent in ICU patients. EBV can be reactivated among immunocompetent patients in ICU, and mortality was higher among patients with EBV reactivation^31,32^. Luca Roncati et al.^33^ reported a case of fatal SARS-CoV-2 coinfection in course of EBV-associated lymphoproliferative disease. In addition to manifesting hyperpyrexia accompanied by dyspnea, the patient also had hepatosplenomegaly. The CT scan showed multiple supra-/subdiaphragmatic lymphadenopathies and a right axillary adenomegaly. This was consistent with our findings that signs and examination results were more severe in the EBV/SARS-CoV-2 coinfection patients. Higher use of corticosteroid, prescribed when patients suffered from CT scan exacerbation or persistent fever exceeding 39℃, was also found in our EBV/SARS-CoV-2 coinfection patients. All of this indicated that EBV reactivation is associated with the severity of COVID-19. We did not find the significant difference in the distribution of patients going to the ICU setting between the EBV/SARS-CoV-2 coinfection patients and SARS-CoV-2 infection alone patients in our study. That may be the small sample of our study.

In this study, we hypothesized that EBV/SARS-CoV-2 coinfection patients may need more time to recovery than the seronegative patients. We analyzed the recovery time between EBV/SARS-CoV-2 coinfection patients and SARS-CoV-2 infection alone patients. The recovery time is a little more in EBV/SARS-CoV-2 coinfection patients, while the difference was not significant. The reason of this negative result might be that most of our included COVID-19 patients were mild cases (85.1%).

Similar to previous study, the typical symptoms on admission of our COVID-19 patients were fever, dry cough, fatigue and myalgia^34,35^, indicating the representativeness of our COVID-19 patients. When clinical symptoms were compared with EBV seropositive antibody, we found that EBV/SARS-CoV-2 coinfection patients had a 3.09-fold risk of having a fever symptom than SARS-CoV-2 infection alone patients.

According to our study, it is recommended to detect EBV in COVID-19 patients with onset time within 2 weeks, suffering from CT scan exacerbation or persistent fever exceeding 39 ℃ or with the above-mentioned changes in laboratory results or going to the ICU setting. EBV co-infected patients may be advised to use corticosteroid.

Our study had several limitations. First, our study was a retrospective design, we could not confirm the time of EBV infection. Second, the sample size in our study was relatively small. Third, most COVID-19 patients did not test the EBV DNA, so we could not assess the viral loads in our study. Forth, because of the small sample and most included patients were mild cases, we could not analyze the statistical associations between anti-EBV antibodies and the mortality of COVID-19.

In summary, our study showed that high incidence of EBV coinfection was in COVID-19 patients. EBV/SARS-CoV-2 coinfection was associated with fever and increased inflammation in COVID-19 patients. EBV reactivation may associated with the severity of COVID-19. The underlying mechanism of how EBV reactivates and affects the COVID-19 needs to be investigated.

## Methods

### Study population

COVID-19 hospitalized patients were enrolled from January 9 to February 29, 2020 at Renmin Hospital of Wuhan University in Wuhan, Hubei province, China. The inclusion criteria in our study were as follows: (1) At least one positive result by real-time quantitative reverse-transcriptase-polymerase-chain reaction (RT-PCR) assay for SARS-CoV-2 when in hospital; (2) Measuring the antibodies against EBV VCA (IgM, IgG), EBV early antigen (EA, IgM) and EBV nuclear antigen (EBNA, IgG); (3) Time of the onset of symptoms to hospital admission less than 2 weeks. Exclusion criteria: (1) In hospital time later than February 29, 2020; (2) Most clinical information were missing. The discharge criteria in our study was according to the diagnosis and treatment protocol for COVID-19 from the National Health Commission of the People’s Republic of China^36^: 1 Afebrile for more than 3 days; 2 Respiratory symptoms significantly improved; 3 Obvious improvement in the radiological abnormalities on chest radiograph; 4 Two consecutive negative SARS-CoV-2 nucleic acid tests at least 24 h intervals. The recovery time was defined as the time from the onset of symptoms to the time of discharge. The ethical committee board of Renmin Hospital of Wuhan University (WDRY2020-K073) approved the study and also waived the need for written informed consent due to the rapid emergence of this infection disease. All methods were performed in accordance with the relevant guidelines and regulations.

### Data collection

The clinical information about the demographic characteristics (i.e., age, sex, comorbidities), symptoms, signs, laboratory tests and CT results, treatments and clinical outcomes (discharge or inpatient) were obtained from the electronic medical records. Two researchers (TC and HLL) recorded the data independently and any differences were resolved by checking the original records. The durations from onset of the first symptom to hospitalization was also recorded. The laboratory tests include the standard blood counts (i.e., white blood cell count, lymphopenia count), blood biochemistry (i.e., alanine aminotransferase, aspartate aminotransferase, Prealbumin), CRP, humoral immunity (i.e., IgG, IgM, C3 and C4) and cellular immunity (i.e., CD3 count, CD4 count and CD8 count).

### Sample collection and pathogens detection

Antibodies against EBV VCA (IgM, IgG), EA IgM and EBNA IgG were detected by Chemiluminesent Immunoassay Assay (CLIA). EBV/SARS-CoV-2 coinfection was defined as SARS-CoV-2 infected patients with VCA IgM positive and SARS-CoV-2 infection was defined as SARS-CoV-2 infected alone patients with VCA IgM negative. Nasopharyngeal swabs were collected from all patients to test for SARS-CoV-2 by real-time RT-PCR according to the same protocol described previously^37^. Other 13 respiratory viruses including the influenza A virus (IFV-A), H1N1, H3N2, influenza B virus (IFV-B), parainfluenza virus (PIV), respiratory syncytial virus (RSV), human metapneumovirus, SARS-CoV, rhinovirus, adenovirus (ADV), Bocavirus, mycoplasma pneumonia and chlamydia were also detected by polymerase chain reaction (PCR) Capillary Electrophoresis Fragment Analysis. Indirect immunofluorescence (IIFA) was used to examine the specific IgM of 9 respiratory pathogens. These pathogens were *legionella pneumophila* (LP), *mycoplasma pneumonia* (MP), *Q fever pneumonia* (COX), *chlamydia pneumoniae* (CP), ADV, RSV, IFV-A, IFV-B and PIV. The antibodies against cytomegalovirus (CMV, IgM and IgG) were also tested.

### Statistical analysis

Frequency variables were reported as numbers and percentages and compared by χ^2^ test or Fisher’s exact. Continuous data were described as median (interquartile range [IQR]), and compared with t test or the Wilcoxon test. The analysis comparing between the EBV seropositive and seronegative COVID-19 patients were performed. Odds ratios (OR) and 95% confidence intervals (CI) were calculated. Statistical analysis was performed with SAS software (SAS 9.3; SAS Institute Inc, Cary, North Carolina, USA). All P values were two-sided and the statistically significant value was < 0.05.

## References

[1] Hui DS (2020). The continuing 2019-nCoV epidemic threat of novel coronaviruses to global health: The latest 2019 novel coronavirus outbreak in Wuhan, China. Int. J. Infect. Dis..

[2] Lu H, Stratton CW, Tang YW (2020). Outbreak of pneumonia of unknown etiology in Wuhan, China: The mystery and the miracle. J. Med. Virol..

[3] World Health Organization. *WHO Director-General's opening remarks at the media briefing on COVID-19—11 March 2020*, https://www.who.int/dg/speeches/detail/who-director-general-s-opening-remarks-at-the-media-briefing-on-covid-19---11-march-2020 (2020).

[4] Yang X (2020). Clinical course and outcomes of critically ill patients with SARS-CoV-2 pneumonia in Wuhan, China: A single-centered, retrospective, observational study. Lancet Respir. Med..

[5] de Wit E, van Doremalen N, Falzarano D, Munster VJ (2016). SARS and MERS: Recent insights into emerging coronaviruses. Nat. Rev. Microbiol..

[6] Sousa H (2011). Epstein–Barr virus in healthy individuals from Portugal. Acta Med. Port..

[7] He H, Wang Y, Wu M, Sun B (2017). Positive Epstein–Barr virus detection and mortality in respiratory failure patients admitted to the intensive care unit. Clin. Respir. J..

[8] Toussirot E, Roudier J (2008). Epstein–Barr virus in autoimmune diseases. Best Pract. Res. Clin. Rheumatol..

[9] Lu JJ (2007). Association of Epstein–Barr virus infection with systemic lupus erythematosus in Taiwan. Lupus.

[10] Hsu JL, Glaser SL (2000). Epstein–Barr virus-associated malignancies: epidemiologic patterns and etiologic implications. Crit. Rev. Oncol. Hematol..

[11] Xu Z (2020). Pathological findings of COVID-19 associated with acute respiratory distress syndrome. Lancet. Respir. Med..

[12] Wang D (2020). Clinical characteristics of 138 hospitalized patients with 2019 novel coronavirus-infected pneumonia in Wuhan, China. JAMA.

[13] Huang C (2020). Clinical features of patients infected with 2019 novel coronavirus in Wuhan, China. Lancet.

[14] Klutts JS, Ford BA, Perez NR, Gronowski AM (2009). Evidence-based approach for interpretation of Epstein–Barr virus serological patterns. J. Clin. Microbiol..

[15] De Paschale M, Clerici P (2012). Serological diagnosis of Epstein–Barr virus infection: Problems and solutions. World J. Virol..

[16] Berkun Y (2009). Infectious antibodies in systemic lupus erythematosus patients. Lupus.

[17] Lehner GF (2020). Correlation of interleukin-6 with Epstein–Barr virus levels in COVID-19. Crit. Care.

[18] Glaser R, Pearl DK, Kiecolt-Glaser JK, Malarkey WB (1994). Plasma cortisol levels and reactivation of latent Epstein–Barr virus in response to examination stress. Psychoneuroendocrinology.

[19] Jaremka LM, Glaser R, Malarkey WB, Kiecolt-Glaser JK (2013). Marital distress prospectively predicts poorer cellular immune function. Psychoneuroendocrinology.

[20] Glaser R, Kiecolt-Glaser JK, Speicher CE, Holliday JE (1985). Stress, loneliness, and changes in herpesvirus latency. J. Behav. Med..

[21] Steven NM (1997). Immediate early and early lytic cycle proteins are frequent targets of the Epstein–Barr virus-induced cytotoxic T cell response. J. Exp. Med..

[22] Liu Y (2020). Clinical and biochemical indexes from 2019-nCoV infected patients linked to viral loads and lung injury. Sci. China Life Sci..

[23] Paolucci S (2020). EBV DNA increase in COVID-19 patients with impaired lymphocyte subpopulation count. Int. J. Infect. Dis..

[24] Simon L, Gauvin F, Amre DK, Saint-Louis P, Lacroix J (2004). Serum procalcitonin and C-reactive protein levels as markers of bacterial infection: A systematic review and meta-analysis. Clin. Infect. Dis..

[25] Xu YH (2020). Clinical and computed tomographic imaging features of novel coronavirus pneumonia caused by SARS-CoV-2. J. Infect..

[26] Shi H (2020). Radiological findings from 81 patients with COVID-19 pneumonia in Wuhan, China: A descriptive study. Lancet. Infect. Dis..

[27] Xiong Y (2020). Clinical and high-resolution CT features of the COVID-19 infection: Comparison of the initial and follow-up changes. Invest. Radiol..

[28] Liu Y, Zhang C, Huang F (2020). Elevated levels of plasma cytokines in COVID-19 reflect viral load and lung injury. Clin. Med..

[29] Zhao D (2020). A comparative study on the clinical features of coronavirus 2019 (COVID-19) pneumonia with other pneumonias. Clin. Infect. Dis..

[30] Mo P (2020). Clinical characteristics of refractory COVID-19 pneumonia in Wuhan, China. Clin. Infect. Dis..

[31] Ong DSY (2017). Epidemiology of multiple herpes viremia in previously immunocompetent patients with septic shock. Clin. Infect. Dis..

[32] Libert N (2015). Epstein–Barr virus reactivation in critically ill immunocompetent patients. Biomed. J..

[33] Roncati L, Lusenti B, Nasillo V, Manenti A (2020). Fatal SARS-CoV-2 coinfection in course of EBV-associated lymphoproliferative disease. Ann. Hematol..

[34] Wang D (2019). Clinical characteristics of 138 hospitalized patients with 2019 novel coronavirus-infected pneumonia in Wuhan, China. JAMA.

[35] Chen N (2020). Epidemiological and clinical characteristics of 99 cases of 2019 novel coronavirus pneumonia in Wuhan, China: A descriptive study. Lancet.

[36] National Health Commission of the People’s Republic of China. *Diagnosis and treatment protocol for novel coronavirus pneumonia *(6rd interim edition), http://www.nhc.gov.cn/xcs/zhengcwj/202002/8334a8326dd94d329df351d7da8aefc2.shtml (2020).

[37] National institute of viral disease control and prevention of the People’s Republic of China. *Primers and probes for detection of the novel coronavirus.* http://ivdc.chinacdc.cn/kyjz/202001/t20200121_211337.html. (2020).

